# Do patients with Autism Spectrum Disorder receive more sedatives in the Emergency Department? A case matched cohort study

**DOI:** 10.1192/j.eurpsy.2025.1450

**Published:** 2025-08-26

**Authors:** S. Das, N. Thomas, R. Arasu

**Affiliations:** 1 Psychiatry, Western Health; 2 Psychiatry, University of Melbourne; 3 Emergency Medicine, Western Health, Melbourne, Australia

## Abstract

**Introduction:**

Adult patients with Autism Spectrum Disorder (ASD) exhibit a range of behaviours that can be disruptive to the medical care of themselves and other patients and as a result, are at higher risk of being sedated. Symptom severity is heterogenous. Some patients are completely non-verbal, some require assistance with basic activities of daily living, and others function independently with only mild difficulties. Roughly two thirds of patients with ASD have a comorbid psychiatric diagnosis, with the most frequent comorbidities being ADHD, anxiety and depressive disorders or intellectual disability. These comorbidities complicate the management of these patients, and may further increase their agitation and distress.

**Objectives:**

The present study aims to determine whether adult patients with ASD who present with acute psychiatric illnesses receive more sedatives in the emergency department.

**Methods:**

43 adult patients with a previous diagnosis of ASD who were referred to the mental health team at a single, large emergency department in metropolitan Victoria over the year of 2021 were identified and matched with an equal number of controls within the same cohort. Sedative medications were converted to diazepam and chlorpromazine equivalent doses.

**Results:**

There were 41.9% female participants among cases and controls. The mean age of patients with ASD was 26.7 (SD = 7.9), which was similar to the mean age of controls of 27.4 (SD = 7.6). The mean hospital length of stay was 13.4 hours (SD 8.6) among cases and 14.0 (SD 7.0) among controls. A higher proportion of patients with ASD received benzodiazepines at 60.5% compared to 46.5% among controls, with a difference that was not statistically significant. A lower proportion of 30.2% of patients with ASD received antipsychotics compared to 44.1% among controls, with a difference that was not statistically significant.

**Conclusions:**

This study shows evidence that patients with ASD are more heavily sedated in the emergency department compared to patients who present similarly but do not have a prior diagnosis of ASD. While the observed increased dose of benzodiazepines was modest, it does represent a potentially increased degree of harm to patients with ASD. We strongly recommends that every attempt should always be made to reduce the use of sedative medications in favour of other techniques for behavioural management. These include verbal de-escalation, reducing sensory stimuli, one-to-one nursing, prompt security presence and the involvement of family or friends. Since patients with ASD are at a higher risk of receiving sedatives, efforts should be made to recognise their patterns of behaviour and difficulty, to understand them and formulate constructive and safe ways to manage their behaviour.

**Disclosure of Interest:**

None Declared

